# Estrogen Signaling in ERα-Negative Breast Cancer: ERβ and GPER

**DOI:** 10.3389/fendo.2018.00781

**Published:** 2019-01-09

**Authors:** Rainer Girgert, Günter Emons, Carsten Gründker

**Affiliations:** Department of Gynecology and Obstetrics, University Medicine Göttingen, Göttingen, Germany

**Keywords:** estrogen signaling, ERα-negative breast cancer, ERα, ERβ, GPER

## Abstract

Estrogen receptors are important regulators of the growth of breast tumors. Three different receptors for estrogens have been identified in breast tumors, two nuclear receptors, ERα and ERβ, and a G-protein coupled estrogen receptor 1 (GPER) that initiates non-genomic effects of estrogens in the cytosol. Recent findings show that the stimulation of cytoplasmic ERα and ERβ also triggers non-genomic signaling pathways. The treatment of breast cancer with anti-estrogens depends on the presence of ERα. About 40% of all breast cancers, however, do not express ERα. One subgroup of these tumors overexpress Her-2, another important group is designated as triple-negative breast cancer, as they neither express ERα, nor progesterone receptors, nor do they overexpress Her-2. This review addresses the signaling of ERβ and GPER in ERα-negative breast tumors. In addition to the well-established EGF-receptor transactivation pathways of GPER, more recent findings of GPER-dependent activation of FOXO3a, the Hippo-pathway, and HOTAIR-activation are summarized.

## Nuclear Estrogen Receptors

Estrogens, in particular 17β-estradiol, exert biological effects in a wide variety of tissues. These effects are dependent on the presence of an appropriate receptor for estrogens. The first estrogen receptor was cloned and characterized in the eighties of the last century from cDNA of the human breast cancer cell line MCF-7 (1). This receptor is a designated estrogen receptor α (ERα) in order to distinguish it from ERβ, which was discovered later. Both receptors are ligand-activated transcription factors that act by binding to DNA in the nucleus. The chromosomal location of the human ERα gene was detected on chromosome 6q25, whereas the gene for human ERβ is located at 14q22-24 (2).

Both estrogen receptors, ERα and ERβ, share common structural features. Five different domains, named A/B, C, D, E, and F, are distinguished in both receptors with varying sequence homology. The N-terminal A/B domain is the most variable and these regions of the human ERα and ERβ share only a <20% identity of the amino acid sequence. The A/B-domain contains the activation function (AF-1) that has been found to be ligand-independent and to possess promoter- and cell-specific activity (3). The C-domain that shares a 95% identity in amino acid sequence in both receptors is responsible for specific DNA-binding to promoters of the target genes. The next domain (D-domain) shares only 30% of homology between the two receptors. It forms a flexible hinge between the DNA-binding C-domain and the E-domain that binds the ligand (17β-estradiol).The D-domain is therefore a designated ligand-binding domain (LBD). This domain harbors the hormone-dependent activation function AF-2 (3).

**Figure 1:**
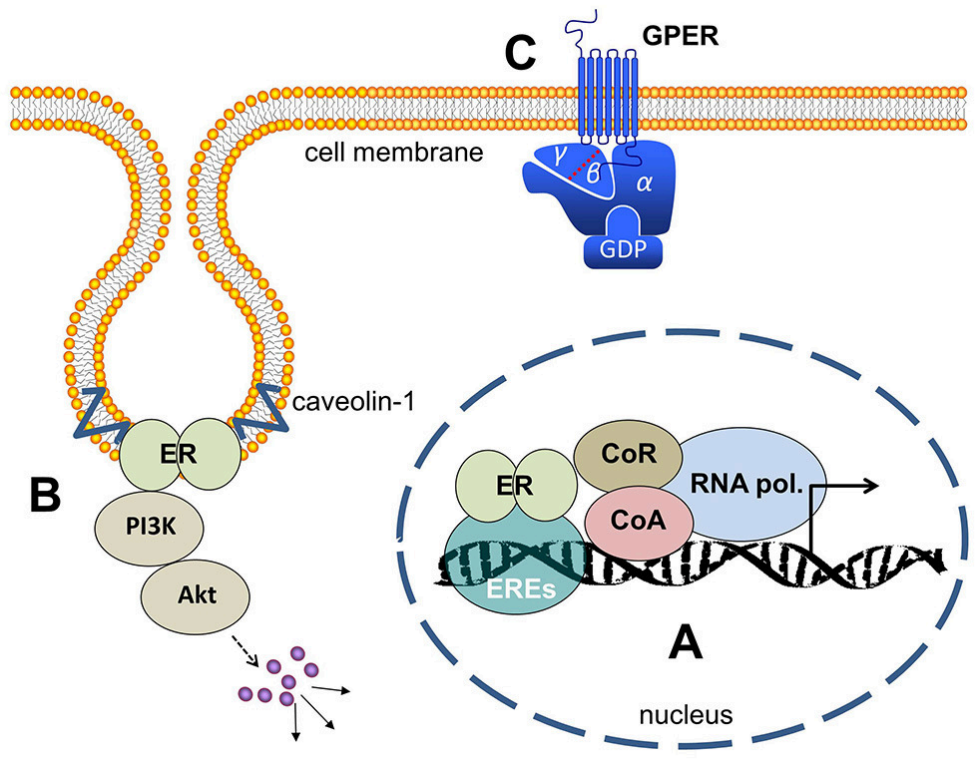
Estrogen receptors **(A)** ER's (either ERα or ERβ-homodimers, or α/β-heterodimers) bind to the ERE (estrogen responsive elements) in the nucleus and activate gene transcription. **(B)** At the cell membrane ER's bound to caveolin and activate cytosolic signaling like PI3-kinase/Akt and MAP-kinases. **(C)** GPER is a membrane-integrated 7-transmembrane receptor activating heterotrimeric G-proteins after estrogen binding and eliciting various signaling pathways as described in the main text. ER, estrogen receptor; ERE, estrogen responsive elements; CoA, co-activator; CoR, co-repressor; PI3K, phosphoinositol-3-kinase; GPER, G-protein coupled estrogen receptor 1; GDP, guanosindiphosphat.

The mechanism of action of ERα and ERβ is quite similar. Before ligand-binding, both receptors are localized in the cytosol of the cells complexed with the chaperon HSP90. The lipophilic ligands, like 17β-estradiol, enter the cells passively by diffusion through the cell membrane. The binding of the ligand leads to a conformational change of the receptors that releases HSP90 from both estrogen receptors into the cytosol. Upon the binding of estrogens, the ERs form dimers and undergo conformational changes that enable them to initiate gene transcription. The estrogen-bound receptors enter the nucleus and bind with their C-domain to the estrogen-responsive elements (ERE), specific DNA-sequences of the promoters of target genes (Figure 1A). The sequence of estrogen-response elements may vary slightly between different target genes, but all have in common a palindromic repeat of nucleotides. For example, the symmetric sequence of the ERE of the vitellogenin gene of Xenopus laevis was determined with the sequence: CAGGTCAnnnTGACCTG (4). Estrogen receptors bound to the promoters initiate the transcription of the respective estrogen receptor target gene into mRNA. To start transcription, a number of cofactors/coactivators and a RNA-polymerase have to bind to the ligand-activated ERs at the promoter. The activated RNA-polymerase synthesizes the m-RNA of the estrogen-dependent target genes and these mRNAs are exported from the nucleus to the ribosomes where they are translated to proteins. These processes are called genomic effects of estrogen and require minutes to hours until they are completed (5). But in addition a number of rapid cytosolic effects of many steroid hormones, like estrogens, androgens and corticosteroids were described in the literature as designated non-genomic effects (Figures 1B,C). These effects are already detectable within seconds or a few minutes. The most important characteristics that distinguish genomic estrogen effects from the fast non-genomic effects is the fact that the genomic effects are inhibitable by inhibitors of RNA-polymerases like cycloheximide and actinomycin D.

## Role of ERα in Breast Cancer

It is well established that the expression of ERα is associated with a clinical response of breast tumors to endocrine therapy, and therefore, it is also associated with a good prognosis of the treated breast cancer patients (6). Three ERα isoforms are mainly distinguished in mammals: full length ERα having a molecular weight of 66 kD and two truncated isoforms, ERα36 and ERα46 with molecular weights of 36 and 46 kD, respectively. These truncated isoforms of ERα lack the first exon of the ERα gene that encodes the activation function (AF-1) (7). Before the discovery of the G-protein coupled estrogen receptor 1 (GPER), ERα36 had been suspected to preferentially mediate most of extra nuclear effects of estrogens. ERα36 is primarily located in the plasma membrane and shows the activation of MAP-Kinases and PI3K. It has been discussed to contribute to endocrine resistance (8). The expression of ERα36 has been detected in breast, endometrial, colorectal, gastric, and hepatic cancer (9). Little is known about the distribution of ERα46, with studies focusing mostly on the vascular effects of ERα46 (10). Observations in MCF-7 breast cancer cells showed that activated ERα also directly interacts with several signaling molecules (11). For example, estradiol induces ERα nuclear export through a mechanism dependent on exportin-1 (XPO1), also known as chromosomal maintenance 1 (CRM1) (11). Several additional findings show that the stimulation of nuclear estrogen receptors located signaling pathways (12).

ERα-negative breast tumors represent 44% of all breast cancer cases. Seventeen percent of these ERα-negative breast tumors additionally overexpress Her-2, the target of trastuzumab and other anti Her-2 directed therapies. The majority of the remaining 81% of ERα-negative breast tumors that do not overexpress Her-2 nor express progesterone receptors is referred to as triple-negative breast cancers. Only two percent of the ERα(-) breast tumors not overexpressing Her-2 are positive for the progesterone-receptor (13). The second nuclear estrogen receptor ERβ is expressed in 44.4% of these triple-negative breast tumors (14).

## The Role of ERβ in Breast Cancer

ERα and ERβ are differently distributed in human breast tissues. The expression of ERα is mainly restricted to nuclei of epithelial cells in lobules and ducts of the healthy breast. ERβ is also expressed in normal breast tissue (15). In addition, ERβ is detectable in myoepithelial cells as well as in surrounding stromal and endothelial cells (16). Increased levels of ERβ in the normal mammary gland were associated with a decreased risk of developing breast cancer (17).

About 60% of all breast cancers that do not express ERα were tested positively for ERβ expression (18). ERβ has been shown to possess a weaker activation function (AF1) than ERα and therefore ERβ is able to repress the transcriptional activity of ERα (19). The importance of ERβ expression on the prognosis of breast cancer is still not clear as ERβ displays different roles depending on the presence or absence of ERα. (20). Early studies supposed that ERβ is a carcinogenic factor in breast tissues (21). However, in more recent studies ERβ is described as a dominant negative regulator of estrogen signaling, since ERβ repressed ERα mediated transcription by forming heterodimers with ERα (22).

A number of isoforms of ERβ were identified as the resultant of alternative splicing of either exon 8 or exon 9 of the ERβ gene (23). The existence of these five different ERβ isoforms complicates the elucidation of the physiological role of ERβ and its involvement in the carcinogenesis of breast cancer (24). Honma et al. (25) analyzed 442 breast cancer patients treated with adjuvant tamoxifen for the impact of ERβ1 expression on overall survival rates. Patients with ERβ1 negative tumors showed a significantly worse overall survival rate than patients with tumors expressing ERβ1. Patients with ERα- and progesterone-receptor negative breast tumors expressing ERβ1 presented with a better prognosis irrespective of whether the tumors expressed Her-2 or not (25, 26).

The other four isoforms of ERβ (β2, β3, β4, and β5) turned out to be unable to bind estrogens (27). ERβ2 is the best studied isoform of all known splice variants of ERβ. It is described as a dominant-negative inhibitor of ERα, as it forms ERβ2/ERα heterodimers that cause a proteasome-dependent degradation of ERα, leading to a suppression of ERα-regulated genes (28). ERβ3 expression was found to be restricted only to the testis. ERβ4 and ERβ5 are truncated transcripts of the ERβ gene that lack ligand binding but these isoforms of ERβ dimerize with ERβ1 and enhance its transcriptional activity in a ligand-dependent manner (29). The best studied isoforms of ERβ are ERβ1 and ERβ2, and their characteristics are summarized in Table 1. The expression of ERβ1 in breast cancer is lower than the expression of ERβ2 and ERβ5 and was detected in 80% of epithelial cells (17). ERβ1 was primarily localized in the nucleus and ERβ2 was detected in the cytoplasm of the tumor cells (30). ERβ1 is able to bind estrogens eliciting transcriptional activity but ERβ2 did not. ERβ1 preferentially forms dimers with ERβ4 and ERβ5 whereas ERβ2 mainly dimerises with ERβ1 (29). ERβ1 binds stronger to the ERE of DNA than ERβ2 and ERβ2 has a higher affinity to the ERE than ERβ5 (23, 31). Several studies investigated the impact of the various ERβ-isoforms on the prognosis of breast cancer. Sugiura et al. observed a better overall survival rate of breast cancer patients that expressed high amounts of either ERβ1 and ERβ2 (32). Similar observations for ERβ1 and ERβ2 were reported from other clinical studies (33, 34). Disease-free survival rates of ERα-negative patients was also improved when tumors expressed both ERβ-isoforms (35). In contrast, Shaaban et al. (36) and Leung et al. (27) reported a longer disease-free survival when ERβ2 was highly expressed in the nucleus of breast cancer cells, and Chantzi et al. observed a poor DFS whenf nuclear ERβ2 was high (37).

**Table 1:** Characteristics of ERβ isoforms ERβ1 and ERβ2.

	**ERβ1**	**ERβ2**
Expression	< β2, < β5	β2
	epithelial 80% (17)nuclear (30)	ductal 32% (17) cytoplasmic (30)
Estrogen binding	yes	no
Transcriptional activity	yes	no
	
Dimers with	ERβ4, ERβ5 (29)	ERβ1 (29)
ERE-binding	>ERβ2 (23)	< ERβ1, > ERβ5 (31)
Impact on prognosis	high ERβ1 ≥ better OS (32) improves DFS in ERα(–) (35) positive ERβ1 better OS (33, 34) high nuc. expr. ≥ shorter OS (30)	high ERβ1 ≥ better OS (32)improves DFS in ERα(–) (35)improved DFS (34)high nuc. expr. ≥ longer DFS (27, 36) but Chantzi et al.: poor DFS (37) high cyt. expr. ≥ shorter OS (30)
	

Nuclear ERβ5 correlated with better OS (36)

*OS, overall survival; DFS, disease free survival*.

Very scarce information is available about the impact of ERβ5 on the outcome of patient. Nuclear ERβ5 correlated with better overall survival rates in breast cancer patients (36).

In patients with stage II breast tumors, the expression of ERβ correlated with longer disease-free survival rates, because the formation of heterodimers of ERα and ERβ reduces the transcriptional activity of ERα (38). In contrast, in node-positive, breast cancer ERβ expression is a biomarker of more aggressive growth as it conveys a higher risk of relapse (39).

ERβ knock-out mice present with quite a normal ductal architecture of the mammary gland ruling out an important role of ERβ in mammary gland development (40). On the other hand, a simultaneous somatic loss of ERβ and the tumor suppressor gene p53 was shown to induce breast tumors in ERβ^(−/−)^/p53^(−/−)^ transgenetic mice (41). The expression of ERβ decreases during breast cancer progression and in higher malignant tumors ERβ-expression is remarkably low (28, 42).

It has been reported that ERβ has opposing effects on the cyclin D1 promoter compared to ERα. 17β-estradiol bound to ERβ repressed cyclin D1 gene transcription and blocked ERα-mediated induction of cyclin D1 when both receptors were present in Hela cells. This is an indication that ERβ is able to modulate the proliferative effects of 17β-estradiol bound to ERα (43). As a further mechanism of the transcriptional activity of estrogens-bound ERα, it was observed that ERα is tethered to the AP-1 site and activates transcription from AP-1 sites of a number of promoters (44). In contrast, 17β-estradiol bound to ERβ had no effects on transcription from AP-1 sites (45).

Besides estrogen receptors, many breast cancer cells also express androgen receptors and become obviously detectable in cells lacking ERα effects of androgens. A comprehensive review on the role of androgen receptors was most recently published by Giovannelli et al. (46).

One particular subtype of ERα-negative breast cancer is triple-negative breast cancer (TNBC) characterized by the lack of ERα and progesterone receptor expression and the absence of Her-2 overexpression. Sixty percent of TNBC tumors were positively stained for ERβ (47). In TNBC, overexpression of the receptor for the epidermal growth factor is one hallmark for increased proliferation of these tumors (48). The expression of ERβ was shown to repress transcription of EGFR (49). In ERα-negative breast tumors, a high nuclear expression of ERβ1 was found particularly in histologically low-grade tumors when ERβ2 was also present in the nuclei. ERβ2 is a marker of early disease recurrence (50). Nuclear ERβ2 is generally associated with poor disease-free survival rates in breast cancer patients. But closer inspections of the function of ERβ lead to the conclusion that its expression seems more likely to have an activity preventing carcinogenesis. In breast cancer tissue the expression of ERβ is lower than in normal breast tissue or benign breast lesions (51). Overexpression of ERβ was shown to downregulate ERα expression by recruiting the corepressor NCoR to the promoter of ERα. In this way, ERβ was able to downregulate the growth-promoting effect of ERα (52). The adenoviral transfer of ERβ induced an increased expression of p21 and p27, causing a cell cycle arrest in G2-phase of cell division (51). In addition, the expression of cyclin D1, a major regulator of the cell cycle entry, has been shown to be inhibited in the presence of ERβ (43).

But ERβ does not only have anti-proliferative effects on breast cancer cells, it also reduces the migration and invasion of these cells, probably by means of repressing the expression of MMP9, necessary for the invasion of the extracellular matrix (53). The agonists of ERβ, liquiritigenin and ERB-041, reduced invasion of TNBC cell lines HCC1806 and HCC1937 into a synthetic extracellular matrix by up to 82%. This effect was accompanied by a significant decrease of the chemokine receptor CXCR4 (54).

## Functions of Membrane-Bound Estrogen Receptor GPER

In addition to the well-characterized genomic responses to estrogen stimulation, some fast actions of estrogens that are elicited at the plasma membrane were described in a great variety of tissues. In 1986, Emons et al. provided data of rapid non-genomic effects of estradiol in pituitary gonadotrophs (55). Using a fluorescent estradiol macromolecular complex (E2-BSA-FITC) the steroid binding to membranes was analyzed on living target cells. Two types of 17β-estradiol-binding sites were detected on hormone-sensitive MCF-7 cells. One was rapidly saturated at low concentrations and a second one had a lower affinity to the macromolecular estradiol complex (56).

For a long time, a membrane-bound ERα was supposed to be responsible for extra-nuclear effects of estrogens (57). The generation of cAMP by adenylyl cyclase is considered to be a process that occurs at the plasma membrane of cells. In MCF-7 breast cancer cells, a 10-fold increase of intracellular cAMP was observed after half an hour of stimulation with 17β-estradiol (58). Estrogen is also able to increase intracellular calcium concentration. In isolated rabbit uteri, estrogen treatment doubled cellular calcium uptake (59). In cultured pituitary cells from adult female rats, the acute negative 17β-estradiol effect on GnRH-induced LH release was found to be mediated via a non-genomic mechanism (60). The authors speculated that the negative effect of 17β-estradiol might be mediated via a modulation of PKC activation. In female rat osteoblasts, 17β-estradiol increased intracellular calcium within the first 2 min of stimulation. This effect was partially inhibited by the inhibitor verapamil giving evidences for an influx of calcium from the extracellular milieu and by thapsigargin, an inhibitor of calcium stores in the endoplasmic reticulum. The initial event leading to increased cytosolic calcium is the hydrolysis of phosphatidylinositol 4,5-biphosphate by phospholipase C generating inositol 1,4,5 triphosphate (IP3) that triggers release from calcium-stores and diacylglycerol. Inhibitors of phospholipase C and pertussis toxin abolished the increase of calcium in the cytosol by 17β-estradiol (61). In addition, tamoxifen, an inhibitor of the genomic effects of ERα was not able to block the rapid increase of Ca2+, IP3 and diacylglycerol. The increased calcium concentration in the cytosol activated the mitogen-activated protein kinase (MAP-Kinase) (62).

For many years, the real nature of the estrogen receptor responsible for the rapid effects of 17β-estradiol remained unknown. First experiments detecting estrogen-binding capacities on endometrial cells were performed, using membrane-impermeable 17β-estradiol coupled to bovine serum albumin (57). On MCF-7, a breast cancer cell line expressing the nuclear estrogen receptor ERα, 17β-estradiol covalently linked to a fluorescein-labeled serum albumin bound saturably with a high affinity to the cell membrane. On MDA-MB-231 cells lacking ERα expression, no such binding sites for 17β-estradiol were detectable. This observation was initially explained by the presence of a membrane-resident ERα that might be responsible for the observed rapid estrogen signaling in breast cancer cells (56). In the Chinese hamster, ovary cell transiently transfected with ERα cDNA binding studies with labeled 17β-estradiol revealed that the receptor is detectable in the nucleus as well as in the cell membrane with almost identical dissociation constants for estradiol, but the receptor number in the cell membrane was only 3% of the nuclear receptor density (63). Using a number of ERα-specific antibodies, it was possible to detect three different epitopes of ERα on GH_3_/B6 rat pituitary tumor cells. It remained unknown how these ERα molecules are fixed at the plasma membrane. With the help of a computer-assisted hydrophobicity analysis of the ERα amino acid sequence, the region between AS 381 and AS 397 was identified as putative membrane-spanning domain of ERα (64).

The real nature of the membrane bound estrogen receptor is now well established. The results of Lieberherr et al. already pointed to an involvement of a receptor coupled to a heterotrimeric G-protein in the non-genomic effects of 17β-estradiol (61). Using differential cDNA library screening, a gene called GPR30, was identified, and later proved to be responsible for many of the non-genomic, membrane-initiated effects of estrogens. GPR30 (Figure 1C) is a 7-transmembrane protein belonging to the large family of G-protein coupled receptors that are embedded into cellular membranes (65, 66). In more recent literature, GPR30 has been renamed to G-protein coupled estrogen receptor 1 (GPER) to better describe this receptor's functionality. Meanwhile, a great number of natural and synthetic GPER-agonists and –antagonists were identified. Some phytoestrogens, like genistein and coumestrol, the anticancer agents 4-hydroxytamoxifen and fulvestrant, the synthetic estrogen, diethylstilbestrol, many pesticides, like DDT and DDE, herbicides, like atrazine and chemical compounds, bisphenol A and nonylphenol are known to be GPER-agonists, apart from 17β-estradiol (67). In addition to the selective estrogen receptor modulator tamoxifen, the complete ERα antagonist Fulvestrant (ICI 182,780) binds to GPER and activates signaling pathways in breast cancer cells, thus leading to the stimulation of proliferation (68).

The synthetic GPER agonist G1 that has a higher affinity to GPER than to ERα is frequently used as a GPER-agonist in experiments analyzing the signaling pathways of GPER. The discovery that 4-hydroxytamoxifen is a GPER-agonist led to the conclusion that GPER may contribute to the emergence of tamoxifen-resistant breast cancer. Ignatov et al. have shown that expression of GPER is in fact increased in breast tumors with acquired tamoxifen resistance (69).

The estrogen estriol has been identified as a natural GPER antagonist (70). A further inhibitor of GPER signaling, the substituted dihydroquinoline G15, was identified in a biomolecular screening of a compound library of small molecules (NIH-MLSMR). It binds to GPER with an affinity of 20 nM. G15 was tested in the GPER expressing breast cancer cells SKBr3 and was able to effectively block calcium mobilization by 17β-estradiol (71). Meanwhile, a compound named G36 was developed as improved antagonists with a higher affinity to GPER (72).

The localization of GPER has been controversially debated in literature for many years. GPER is known to be a G-protein coupled with the 7-transmembrane receptor this fact implies that GPER is an integral membrane protein. However, the expression of a fusion protein of GPER and green fluorescent protein in monkey kidney cells (COS7) pointed to an intracellular localization of GPER as GPER co-localized with the endoplasmic marker protein KDEL (73). Otto et al. reported that GPR30 localizes to the endoplasmic reticulum in COS7- and HEK293 cells but is not activated by estradiol (74).

On the other hand, in HeLa-cells transfected with an N-terminally FLAG-tagged GPER, expression of this protein was detected in the plasma membrane (66). The localization of GPER in the plasma membrane was confirmed through the experiments conducted by Sanden et al. (75). They showed that in Mardin-Darby canine kidney cells and in the human breast cancer cell line T47D, 17β-estradiol stimulated cAMP synthesis in a GPER dependent manner, a process defined as a plasma membrane event. In addition, they observed a GPER-dependent recruitment of β-arrestin2 to the plasma membrane of estradiol-stimulated cells. The staining of living cells showed that GPER also localized to cytoplasmic intermediate filaments that contained cytokeratins, CK7 and CK8, but not to the endoplasmic reticulum (75). This subcellular distribution of GPER between plasma membrane and cytoplasm was confirmed in the breast cancer cell line MCF-7 and in sections of a number of breast tumors (76).

In an excellent experimental series, Broselid et al. (77) highlighted the mechanisms of how GPER is localized to the plasma membrane. In HEK293 cells GPER forms a complex with MAGUK and AKAP5, where both proteins are associated with the plasma membrane. This binding was shown to depend on a C-terminal PDZ motive present in GPER. An N-terminally FLAG-tagged GPER-construct lacking this PDZ motive omitted the binding of GPER to the plasma membrane and disrupted 17β-estradiol dependent GPER signaling (77).

## Signal Transduction of GPER

**Figure 2:**
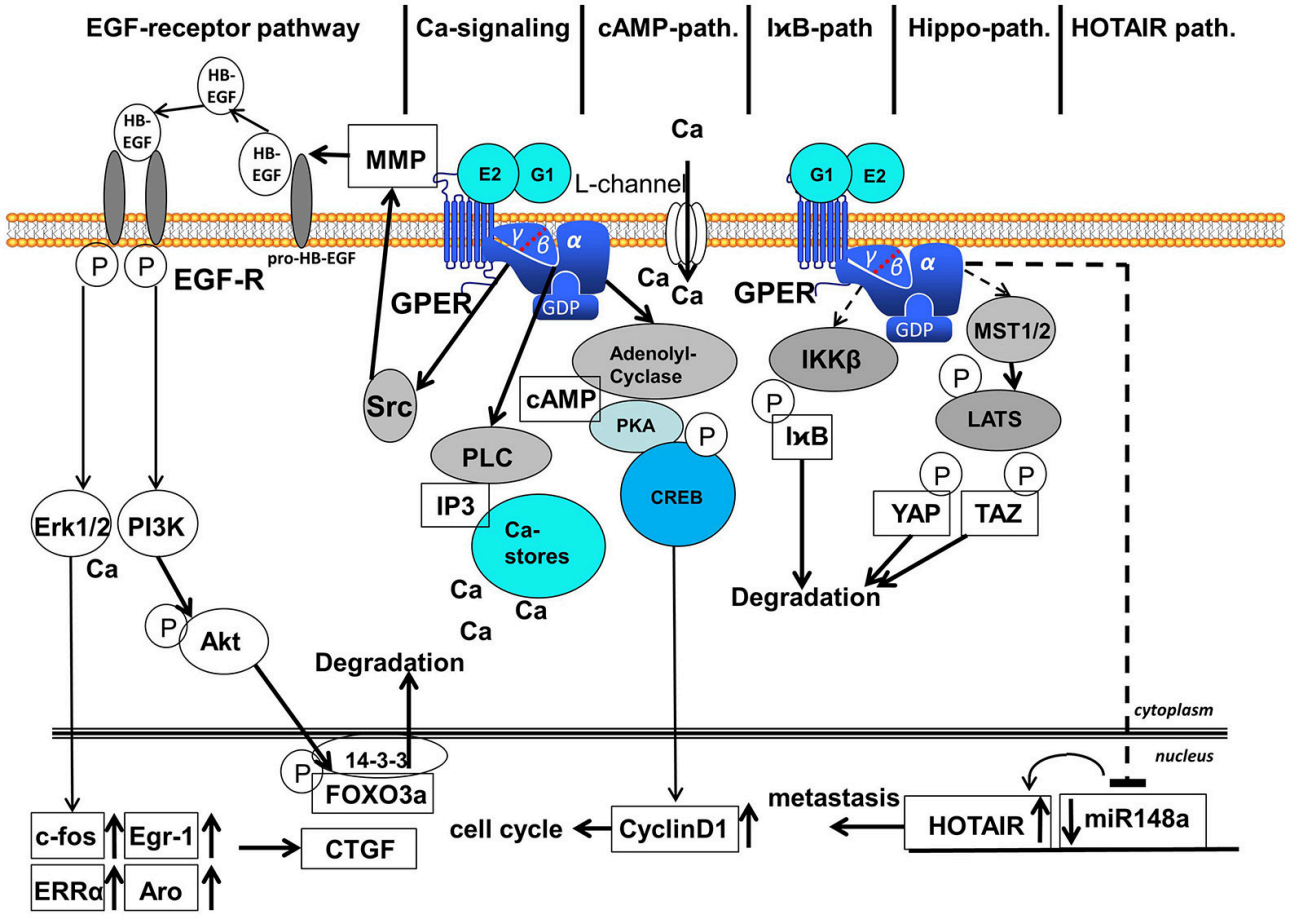
Signaling pathways activated after binding of E2 and G1 to GPER. Six different signaling pathways are distinguished: (1) EGF-receptor-pathway, (2) calcium-signaling, (3) cAMP-pathway, (4) IχB-pathway, (5) Hippo-pathway, and (6) HOTAIR-pathway. EGF, epidermal growth factor; MMP, matrix-metalloproteases; GPER, G-protein coupled estrogen receptor 1; Erk1/2, extracellular regulated kinase 1 and 2; PI3K, phosphoinositol-3-kinase; PLC, phospholipase C; IP3, inositol-triphosphate; cAMP, cyclic adenosine-monophosphate; PKA, protein kinase A; CREB, cAMP-responsive element binding protein; I
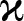
B, inhibitor of 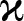; IKKβ, inhibitor of 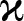 kinase β; MST1/2, mammalian sterile 20-like 1 and 2; LATS, large tumor suppressor; YAP, *yes*-associated protein; TAZ, transcriptional coactivator with PDZ binding motif; HOTAIR, HOX-transcript antisense intergenic RNA; FOXO3a, forkhead box 3a; CTGF, connective tissue growth factor; After binding of 17β-estradiol or G1 to GPER various signaling pathways are activated in the cytosol. Ligand binding to GPER leads to the detachment of the βγ-subunit of heterotrimeric G-proteins. The EGF-receptor pathway starts with the activation of the kinase Src by βγ-G-protein that activates MMPs, liberating EGF from heparin-bound EGF. EGF binding to EGF-receptor leads to auto-phosphorylation of the EGF-receptor that activates MAP-kinase Erk1/2 and PI3-kinase. Activated Erk induces transcription of c-fos, Egr-1, ERRα and aromatase in the nucleus followed by the induction of CTGF. PI3-kinase is also activated by the phosphorylated EGF-receptor and phosphorylates the kinase Akt that phosphorylates transcription factor FOXO3a in the nucleus that is subsequently exported to the cytosol, where FOXO3a is degraded. The Calcium signaling pathway starts with the released α-subunit of heterotrimeric G-proteins that activates phospholipase C. PLC cleaves Phosphatidylinositol-4,5-bisphosphat to diacylglycerol and IP3. IP3 releases calcium ions from cytosolic calcium-stores. Ca^2+^ activates several enzymes in the cytosol among other things Ca^2+^ additionally activates Erk1/2. Binding of 17β-estradiol to GPER also opens calcium L-channels in the plasma membrane by a yet unsolved mechanism. The released α-subunit of the heterotrimeric G-protein also activates the adenolyl-cyclase (AC) in the cytosol. cAMP generated by AC activates protein kinase A (PKA) that phosphorylates the cAMP response element binding protein (CREB). Phosphorylated CREB binds as a transcription factor to promoters of genes containing a cAMP response element, for example cyclinD1, that supports the progress of the cell cycle. Less elucidated are the I
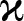
B-pathway, Hippo-pathway and HOTAIR-pathway represented in this figure by hatched arrows. In the course of the I
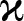
B-pathway IKKβ is activated that phosphorylates I
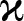
B, an inhibitor of NF-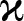
B. Phosphorylated I
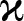
B is degraded via the ubiquitination pathway allowing NF
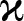
B action. In the course of the Gα-protein dependent Hippo-pathway MST1/2 are activated that phosphorylates LATS, a kinase phosphorylating the transcription factors YAP and TAZ that are subsequently degraded. In the HOTAIR-pathway expression of miR148a is inhibited in a Gα-dependent manner, but the intermediate steps leading to transcription of this microRNA are not completely elucidated, in this figure exemplified by a hatched arrow. Suppression of miR148a expression leads to an increased expression of HOTAIR that finally supports metastasis.

Many different signal transduction pathways are activated by GPER after the binding of 17β-estradiol and are described in detail below (Figure 2).

### Calcium-Signaling Induced by GPER

Calcium ions are known as essential intracellular messengers the regulate many cellular processes, like enzyme activity, muscle contraction or hormone secretion (78). Even the rapid increase of intracellular calcium concentration by 17β-estradiol that has initially been attributed to membrane-resident ERα has been shown to depend on the presence of GPER. Cos7-cells known to be negative for ERα were transfected with GPER. In these transfected cells, a similarly strong calcium mobilization was evoked 20 s after stimulation with 0.1 nM 17β-estradiol as in Cos7 cells expressing ERα after transfection with ERα-GFP (73). This observation pointed to the presence of a third estrogen receptor different from the previously described nuclear receptors, ERα and ERβ. But GPER does not only affect calcium mobilization from intracellular stores. In rat myometrium cells the GPER-specific agonist G1, induced a membrane depolarization by opening the nifedipine sensitive, L-type calcium channels in the cell membrane that allowed calcium influx from the extracellular environment (79).

### Activation of MAP-Kinases

The signaling events triggered by the binding of 17β-estradiol to GPER were analyzed in detail by Filardo et al. (68). They observed the activation of MAP-kinase Erk after exposure to 17β-estradiol in a number of human breast cancer cell lines exhibiting different ERα expression profiles. Even in breast cancer cell line SKBr3 that expresses neither ERα nor ERβ, they determined a 6-fold increase of Erk phosphorylation after the stimulation of these cells with 1 nM 17β-estradiol for 5 min. On the other hand, in the cell line MDA-MB-231 that lacks expression of GPER, an increase of Erk phosphorylation was not induced by estrogen stimulation. After transfection of MDA-MB-231 cells with a vector containing the GPER gene, these cells became estrogen-responsive and Erk phosphorylation was clearly detectable following stimulation with 17β-estradiol. In the same experimental setting, the authors observed that this estrogen-mediated, GPER-dependent, Erk activation was sensitive to pertussis toxin pointing to an involvement of the βγ-subunits of heterotrimeric G-proteins. Additional experiments, using src family tyrosine kinase inhibitor PP2 revealed that this pertussis toxin sensitive Erk activation by 17β-estradiol requires the activity of the cytosolic kinase src. As the missing link between estrogen-activated src and the E2-activated MAP-Kinase Erk, the necessity of cleavage of heparin-bound EGF was recognized (68). From earlier work by Prenzel et al. (80), it is known that stimulation of G-protein coupled receptors in Rat-1 fibroblasts with endothelin-1 or thrombin activates membrane-bound metalloproteinases that release EGF from heparin-bound EGF (HB-EGF) to stimulate EGF-receptor, finally leading to Erk activation (68, 81).

### Induction of Transcription Factors by Estrogens via GPER

In the nucleus, activated Erk increases expression of proliferation-inducing genes, like c-fos, and cyclinD1. The induction of c-fos and cyclinD1 expression by 17β-estradiol has particularly been shown in ERα/ERβ negative cell line SKBr3 and in triple-negative breast cancer cell lines (82, 83). A microarray analysis of gene expression in the GPER-expressing breast cancer cell line SKBr3, after stimulation with estradiol and with the GPER agonist tamoxifen, revealed that the connective tissue growth factor CTGF was induced nearly 10-fold and the deletion of GPER by siRNA prevented the induction of CTGF. In addition, several transcription factors, including c-fos and Egr1, were found to be upregulated by 17β-estradiol and tamoxifen using gene expression profiling of the RNA of the stimulated cells. From these experiments, CTGF appears to be a target of these transcription factors (84). CTGF is more prominently known as mediator of tissue fibrosis but has also been reported to be involved in the tumorigenesis of breast cancer (85).

### Activation of PI3-Kinase

Besides Erk activation, the transactivation of the EGF-receptor by estradiol also activates PI3-kinase. The stimulation of an ERα-negative endometrial carcinoma cell line (Hec50) with 17β-estradiol or the GPER-specific agonist G1 was able to increase PI3-Kinase activity (86).

Downstream of PI3-kinase, the activation of the kinase Akt phosphorylates the transcription factor FOXO3a, a member of the forkhead-box gene family that is recognized as a tumor suppressor gene (87). In case of reduced energy supply as it is common in large solid tumors FOXO3a also acts as important inhibitor of cell cycle progression and cellular proliferation (88, 89).

### Degradation of FOXO3a After Activation of GPER

The phosphorylated FOXO3a protein is subsequently bound to the nuclear export protein 14-3-3 and is then ubiquitinated and degraded by the proteasome complex in the cytosol. Nuclear exclusion of FOXO3a by activation of Akt increases cellular survival. Treatment of MCF-7 breast cancer cells with the selective GPER agonist G1 was shown to lead to an inactivation of FOXO3a (90).

In addition, the pro-survival effect of 17β-estradiol on breast cancer cells through GPER involves expression of SIRT1, a putative tumor suppressor, being a coactivator of FOXO3a, as described below. In the ERα-negative breast cancer cell line SKBr3, estradiol and GPER agonist G1 increased SIRT1 expression (91).

In triple-negative breast cancer cells MDA-MB-453 and MDA-MB-231, degradation of FOXO3a has been shown to reduce the expression of Fas-ligand, p27^kip^ and bim, thereby preventing the induction of apoptosis. In addition, a second signaling pathway leading to FOXO3a inactivation has been discovered to be independent of Akt.

### Inhibition of NFκB Following GPER-Activation

In some breast tumors devoid of p-Akt, cytoplasmic localization of FOXO3a correlates with the expression of IKKβ and is associated with poor survival rates. Phosphorylation of IκB by IκB kinase leads to the degradation IκB by the ubiquitin proteasome and thereby prevents the proliferative and antiapoptotic activity of NFκB. (92). It has further been shown that stimulation of GPER by the agonist G1 suppresses EMT of the TNBC cell line MDA-MB-231 by the down-regulation of NFκB signaling (93).

### Activation of Adenylyl Cyclase

Parallel to the βγ-G-protein dependent signaling pathways of GPER, it has also been demonstrated that stimulation of GPER causes activation of the Gαs protein finally leading to adenylyl cyclase activation and cAMP accumulation in the estrogen stimulated breast cancer cells (94, 95). An elevation of intracellular cAMP by 17β-estradiol was observed in the breast cancer cell line SK-Br3 and in MDA-MB-231 cells transfected with the GPER gene. Both cell lines lack the expression of classical ERα. Surprisingly, it was also shown that the generation of cAMP in these cells attenuated the activation of Erk-1/2, thereby limiting the effects of 17β-estradiol on the EGF-receptor/Erk signaling axes (94).

Further downstream of GPER signaling the elevated cAMP activates protein kinase A. PKA phosphorylates the transcription factor CREB, leading to the transcription of genes possessing a cAMP responsive element in their promoter (96, 97). Phosphorylated CREB binds to the promoters of cyclinD1 and cyclinD2 and induces their transcription. CyclinD1 and cyclinD2 are both involved in the progression of cell cycle (82, 96).

### Activation of Hippo-Signaling

For example, estrogen leads to a GPER-dependent activation of the Hippo-signaling pathway. The Hippo-pathway is involved in the control of organ size, cell proliferation, and tumor development. In basal cell carcinoma, YAP clearly increased the expression of Cyr61 (CCN1) and the connective tissue growth factor (CCN2) (98), building a bridge between activation of GPER and induction of CTGF expression, as observed by Pandey et al. (84) in SKBr3 breast cancer cells.

The Hippo-pathway involves the yes-associated protein 1 (YAP) and TAZ, a transcriptional coactivator with a PDZ-binding domain and the transcription factors of the TEA domain family (TEAD). Tumor suppression by the Hippo-pathway was reported to depend on the activity of Gαq-11, PLCβ, and Rho/ROCK. The Hippo/YAP/TAZ pathway was found to be a downstream branch of GPER signaling playing an important role in breast tumorigenesis (99).

Activation of Hippo pathway starts with MST1/2 (mammalian sterile 20-like 1), a kinase that activates LATS1/2 kinases (Large tumor suppressor). LATS1/2 inactivates the transcription factors YAP and TAZ by phosphorylation. Phosphorylated YAP and TAZ interact with the protein 14-3-3 and are retained in the cytoplasm where they are degraded by the proteasome complex (99).

### Induction of Hotair

In ERα-positive as well as in triple-negative, breast cancer cell lines' expression of HOTAIR was induced by 17β-estradiol as another GPER-dependent signaling event (100). HOTAIR (HOX transcript antisense intergenic RNA) is one of many long non-coding RNAs. It is upregulated in tumors of many different cancers, including breast cancer. HOTAIR is involved in the control of apoptosis, metastasis and DNA-repair. In breast cancer metastasis HOTAIR is overexpressed more than 100-fold (101). ERα-negative tumors with a high expression of HOTAIR preferentially use the lymphatic vessels to spread to secondary sites. High levels of HOTAIR expression were associated with worse prognoses. The influence of HOTAIR expression on overall survival was analyzed in a cohort of 132 breast cancer patients. In ERα-negative breast cancer patients with high HOTAIR expression, the overall survival rate at the 100th month after diagnosis was only 46.4% compared to 62.8% in patients with low expression of HOTAIR. In contrast, in ERα-positive breast tumors no correlation was observed between overall survival and HOTAIR expression (p = 0.41) (102). HOTAIR binds to promoters of silenced metastasis suppressor genes where it recruits the lysine-specific demethylase 1 (LSD1), finally leading to the induction of cancer metastasis (103). GPER-dependent migration of TNBC cell lines MDA-MB-231 and BT549 was reversed by deletion of HOTAIR in these cell lines. The upregulation of HOTAIR was shown to be achieved by the GPER-dependent suppression of microRNA miR148a (100).

### Particular Role of GPER in Triple Negative Breast Cancer

In TNBC, GPER is frequently expressed very strongly and high GPER expression in this subgroup of breast cancer was found to correlate with increased recurrence. After a 36-month follow-up, 90.5% of the TNBC patients with low GPER expression were still alive, whereas in the cohort with high GPER expression only 77.8% of the patients survived after this time period (104). A knock-down of the GPER expression, using GPER-specific siRNA, was shown to prevent 17β-estradiol-dependent growth stimulation of triple negative breast cancer cell lines. In the TNBC cell lines treated with GPER-siRNA, the activation of c-src and transactivation of EGFR by 17β-estradiol and tamoxifen were completely inhibited (82). The results of these experiments provide further evidence that the non-genomic effects of 17β-estradiol, including activation of c-Src, phosphorylation of the EGF-receptor, activation of the MAP-kinase Erk1, and increased expression of c-fos are dependent on the presence of GPER.

## Therapeutical Options Targeting GPER in Triple Negative Breast Cancer

### Pharmacological Inhibition of GPER

Several pharmacological inhibitors of GPER have been identified. Using estriol, a natural estrogen, GPER signaling was successfully prevented in the ERα-negative breast cancer cell line SKBr3 (70). In the triple-negative breast cancer cell lines, HCC1806 and HCC70, activation of Src-kinase and EGF-receptor. The induction of c-fos by 17β-estradiol was also inhibited after pre-treatment of the cells with 10^−4^ M estriol (105).

In TNBC, the synthetic GPER antagonist G15 proved to be less effective in inhibiting GPER signaling than estriol (unpublished results).

### Suppression of GPER Expression

- Inhibition of EGF-receptor
  A different approach to inactivate GPER in triple-negative breast cancer is the suppression of GPER expression. In many triple-negative breast tumors, an overexpression of the EGF-receptor (Her-1) has been detected instead of an overexpression of Her2 (106). EGF is able to induce the expression of GPER in estrogen receptor negative breast cancer cells, thus facilitating a growth stimulatory effect of 17β-estradiol even in breast cancer cells lacking ERα expression (107). The fact that 17β-estradiol trans-activates the EGF-receptor via GPER and Src and so increases the expression of GPER leads to a positive feedback loop that boosts the induction of proliferation by 17β-estradiol in triple-negative breast tumors. The treatment of TNBC cells with gefitinib, an inhibitor of the tyrosine-kinase of the EGF-receptor, reduced GPER expression by up to 85%. This reduction of GPER expression successfully prevented all signaling events downstream of GPER as the activation of Src-kinase, the transactivation of the EGF-receptor, and the induction of c-fos expression by 17β-estradiol (108).
- (b) Inhibition of growth hormone receptor
  Growth hormone (GH) is another factor involved in the regulation of GPER expression. Antagonists of growth hormone-releasing hormone were shown to suppress *in vivo* growth of TNBC (109). Somavert (Pegvisomant) is a specific inhibitor of the GH-receptor that is already clinically applied in the treatment of acromegaly (110). In triple-negative breast cancer cell lines, the expression of GPER was reduced by dose-dependent treatment with Somavert. The treatment of MDA-MB-453- and HCC1806 cells with 1 μM Somavert reduced GPER-dependent p-src and EGF-receptor activation by almost 50% and induction of cyclinD1 and aromatase by estradiol was completely prevented by pretreatment of the cells with Somavert (97). The inhibition of GPER expression is a promising therapeutic intervention for TNBC.

Additional downstream pathways activated by GPER include PI3K (73, 86), PKCε (111), and voltage-gated sodium channels (112).

It has been shown that in many different cellular systems a multitude of signaling pathways are activated by 17β-estradiol or other ligands of GPER that are responsible for the induction of proliferation or increased metastasis as shown in Figure 2. In brief, signaling pathways dependent on binding of 17β-estradiol to GPER are classified as (1) EGF-receptor pathway, (2) calcium-signaling, (3) cAMP-pathway, (4) I
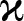
B-pathway, (5) Hippo-pathway, and (6) HOTAIR-pathway.

After the binding of 17β-estradiol or G1 to GPER, various signaling pathways are activated in the cytosol. Ligand binding to GPER leads to the detachment of the βγ-subunit of heterotrimeric G-proteins. The EGF-receptor pathway starts with the activation of the kinase Src by βγ-G-protein that activates MMPs, liberating EGF from heparin-bound EGF. EGF binding to EGF-receptor leads to auto-phosphorylation of the EGF-receptor that activates MAP-kinase Erk1/2 and PI3-kinase. Activated Erk induces transcription of c-fos, Egr-1, ERRα and aromatase in the nucleus followed by the induction of CTGF. PI3-kinase is also activated by the phosphorylated EGF-receptor and phosphorylates the kinase Akt that phosphorylates the transcription factor FOXO3a in the nucleus, which is subsequently exported to the cytosol, where FOXO3a is degraded. The Calcium-signaling pathway starts with the released α-subunit of heterotrimeric G-proteins that activates phospholipase C. PLC cleaves Phosphatidylinositol-4,5-bisphosphat to diacylglycerol and IP3. IP3 releases calcium ions from cytosolic calcium-stores. Ca^2+^ activates several enzymes in the cytosol, among other things, and activates Erk1/2. The binding of 17β-estradiol to GPER also opens calcium L-channels in the plasma membrane by a yet unsolved mechanism. The released α-subunit of the heterotrimeric G-protein also activates the adenolyl-cyclase (AC) in the cytosol. cAMP generated by AC activates protein kinase A (PKA) that phosphorylates the cAMP response element binding protein (CREB). Phosphorylated CREB binds as a transcription factor to promoters of genes containing a cAMP response element, for example cyclinD1, which supports the progress of the cell cycle. Less elucidated are the I
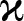
B-pathway, Hippo-pathway and HOTAIR-pathway represented in this figure by hatched arrows. In the course of the I
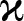
B-pathway, IKKβ is activated and phosphorylates I
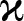
B, an inhibitor of NF-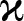
B. Phosphorylated I
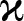
B is degraded via the ubiquitination pathway, allowing NF
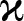
B action. In the course of the Gα-protein-dependent Hippo-pathway, MST1/2 are activated and phosphorylate LATS, a kinase phosphorylating the transcription factors YAP and TAZ, which are subsequently degraded. In the HOTAIR-pathway, the expression of miR148a is inhibited in a Gα-dependent manner, but the intermediate steps leading to transcription of this microRNA are not completely elucidated, in this figure exemplified by a hatched arrow. The suppression of the miR148a expression leads to an increased expression of HOTAIR that finally supports metastasis.

## Conclusion

Apart from estrogen receptor ERα that was discovered first two further estrogen receptors, ERβ and GPER are known to be important for the hormonal control of breast cancer. The complete elucidation of the function of ERβ in breast cancer is hampered by the diversity of isoforms of ERβ identified. There are still a lot of efforts to be done to clarify the impact of the various isoforms of ERβ on the transcription of target genes of estrogens in particular how the many possible heterodimers act in breast cancer cells.

For GPER, the estrogen receptor that is commonly accepted to be responsible for the extra-nuclear, non-genomic effects of estrogens a large number of signaling pathways were identified in recent years. In addition to the well-known transactivation of the EGF-receptor that leads to the activation of the MAPK-kinase- and the PI3-kinase pathway a number of further signaling events have been described to be induced by the binding of estrogens to GPER. (1) The inactivation of the transcription factor FOXO3a via the kinase Akt. (2) The activation of the Hippo-pathway that induces CTGF expression followed by enhanced metastasis. (3) Induction of HOTAIR expression that also increases the metastatic potential of breast cancer cells. The knowledge about these GPER-dependent signaling pathways will promote the development of new targeted therapies. In particular for triple negative breast tumors that strongly express GPER such therapies are urgently needed.

## Author Contributions

RG and CG drafted the manuscript. GE critically revised the manuscript. All authors read and approved the final version.

### Conflict of Interest Statement

The authors declare that the research was conducted in the absence of any commercial or financial relationships that could be construed as a potential conflict of interest.
